# Liver pseudoaneurysm mimicking haemangioma: a multimodal imaging trap and embolization pitfall

**DOI:** 10.1186/s42155-025-00613-3

**Published:** 2025-11-08

**Authors:** Mohamed Mostafa Fouad, Gaetan Davout, Aya E. Ahmed, Alexis Quirantes, Norhane Chadli, Olivier Chevallier, Romaric Loffroy

**Affiliations:** 1 Department of Vascular and Interventional Radiology, Image-Guided Therapy Center, François-Mitterrand University Hospital, Dijon, France; 2 Department of Diagnostic and Interventional Radiology and Molecular Imaging, Ainshams University, Cairo, Egypt; 3 Department of Radiology, University Hospital of Constantine, Constantine, Algeria

**Keywords:** Hepatic artery pseudoaneurysm, Hepatic haemangioma, Embolization, Computed tomography, Angiography, Magnetic resonance imaging

## Abstract

**Background:**

Hepatic artery pseudoaneurysms (HAP) and hepatic haemangiomas (HH) may present with indistinguishable imaging characteristics, particularly when clinical history favors one diagnosis over the other. Primary imaging alone may be insufficient for definitive differentiation. This case highlights the importance of further non-invasive imaging modalities in avoiding unnecessary invasive procedures if clinical condition allows.

**Case presentation:**

A 55-year-old patient presented with abdominal trauma after a fall. Computed tomography (CT) revealed a grade III liver laceration with a hyper vascular lesion near the right hepatic artery, initially suspected to be a HAP. Trans-arterial embolization (TAE) was planned, and selective catheterization was performed. However, angiography showed no pseudoaneurysm filling but rather features suggestive of a haemangioma, leading to the abortion of the procedure. Subsequent magnetic resonance imaging (MRI) confirmed a flash-filling HH. The patient remained stable, with no haemorrhagic complications or need for further intervention.

**Conclusion:**

In emergencies, recognizing imaging features distinguishing haemangiomas from pseudoaneurysms is crucial to avoid unnecessary invasive procedures, especially in stable patients, using accurate non-invasive tools like CT or MRI.

**Graphical Abstract:**

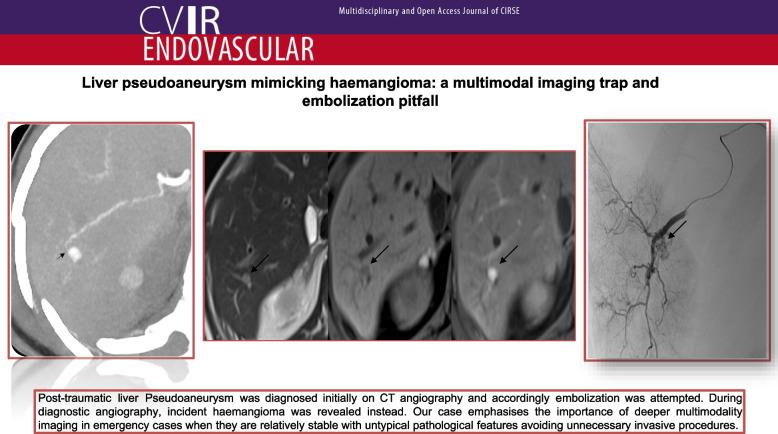

## Background

Hepatic artery pseudoaneurysms (HAPs) and hepatic haemangiomas (HHs) can exhibit overlapping imaging features, especially when clinical history biases interpretation toward one diagnosis over another [1]. In many cases, primary imaging—as well as contrast-enhanced computed tomography (CT) or ultrasound—may be insufficient to reliably differentiate between these vascular lesions [2]. This case underscores the critical role of additional non-invasive imaging modalities, particularly contrast-enhanced magnetic resonance imaging (MRI), in accurately characterizing hepatic lesions. When the patient’s clinical condition is stable, employing advanced imaging techniques can help prevent unnecessary and potentially harmful invasive procedures, ensuring appropriate and safe patient management [3].

## Case presentation

**Fig. 1:**
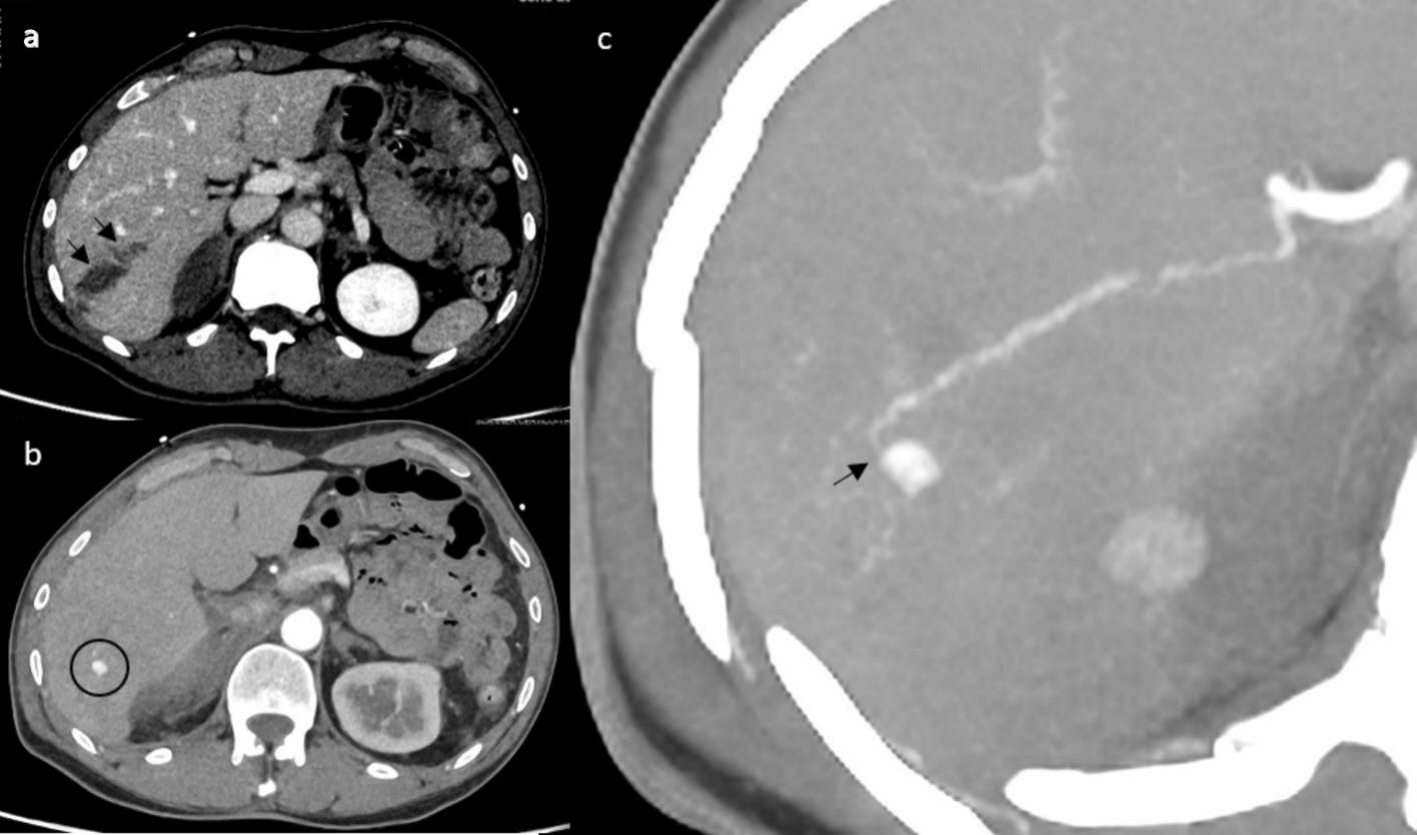
**a** Axial CT tri-phasic of the abdomen shows an irregular linear/branching area of hypoattenuation representing laceration (black arrows) in the hepatic segment VI in portal phase. **b** A query hyper-enhancing lesion measuring about 7 mm is seen in segment VI suspected to be a pseudoaneurysm in arterial phase (black circle). **c** Axial CT Angiography of the abdomen with maximum intensity projection (MIP) confirms the previously noted hyper enhancing lesion with close relation with a branch from the right hepatic artery (black arrow)

A 55-year-old patient was admitted to the trauma intensive care unit after sustaining abdominal and thoracic trauma following a fall of approximately 1.50 m. On admission, fast ultrasound (US) was done and was suspicious of a liver laceration. Additionally, laboratory tests showed elevated liver enzymes (aspartate aminotransferase (AST) at 227 IU/L and alanine aminotransferase (ALT) at 173 IU/L) in addition to a relatively stable hemoglobin level of 13.6 g/dL, without associated evident cholestasis. Initial abdominopelvic CT with contrast imaging (triphasic CT with slice thickness of 5 mm) revealed an AAST grade III liver laceration in the right lobe, with a subcapsular hematoma and some retroperitoneal hematoma with no active bleeding. Additionally, just under and in close proximity to the laceration, a 7 mm spontaneous isodense homogenously hyper-vascular lesion had a close relation to a branch from the right hepatic artery (Fig. 1). According to clinical history and CT presentation, it was diagnosed as HAP.

**Fig. 2:**
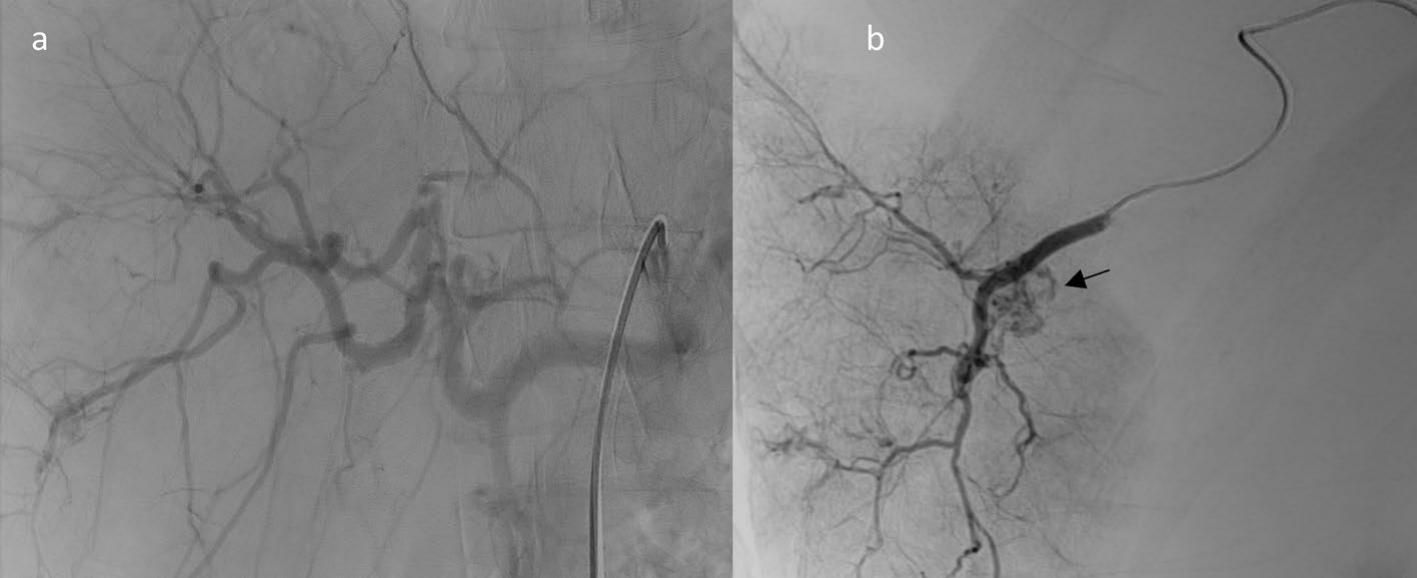
**a** Digital subtraction angiography images with bone and fluoroscopic after catheterization of the CA. **b** Selective catheterization of the RHA showing typical appearance of a flash filling haemangioma arising from the RHA (black arrow)

A follow-up CT scan was advised at 48 h confirming the persistence of the vascular lesion within the hepatic injury, indicating its endovascular trans-arterial embolization (TAE). The procedure started with femoral access using a 6 French sheath through a Seldinger technique. Afterwards, a 5 French Cobra catheter and a hydrophilic guide wire of 0.035 inch and 180 cm long were used to catheterize the celiac artery (CA). Moreover, a super selective catheterization of the right hepatic artery (RHA) was achieved using a microcatheter of 2.7 French. However, a selective angiography did not reveal a pseudoaneurysm contrast filling but a heterogeneous uptake and washout of the vascular lesion indicating more HH (Fig. 2). Finally, the procedure was aborted without embolizing the lesion.

**Fig. 3:**
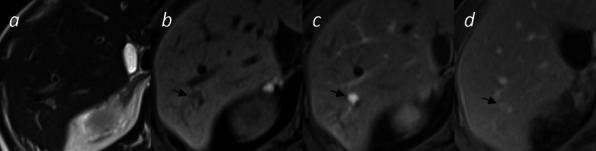
Axial MRI with T2 weighting (**a**), T1 fat suppression before (**b**), and after contrast at arterial phase (**c**) delayed phase—5 min (**d**). T2 hypersignal, T1 hypo signal, and prompt enhancement confirmed the diagnosis of the flash filled haemangioma in the hepatic segment VI (black arrows)

For diagnosis confirmation, a hepatic dynamic MRI was performed, showing T2 hyperintensity, T1 hypo intensity with diffusion restriction, and classically T2 shine through effect on ADC. Furthermore, a homogenous arterial filling with no washout, confirming the diagnosis of hemangioma with flash filling features (Fig. 3). The patient’s clinical course was favorable, with no hemorrhagic complications or need for further therapeutic intervention.

## Discussion

Our case report emphasizes that hepatic artery pseudoaneurysm (HAPs) and hepatic hemangioma (HHs) can exhibit similar imaging characteristics, making differentiation challenging, especially when clinical history favors one diagnosis over the other and the lesion size is small [1]. Thus, primary imaging alone may not always be sufficient for a definitive diagnosis [2]. This underscores the importance of additional accurate, non-invasive modalities such as CT or MRI, which can delineate the lesion’s features and distinguish it from other hepatic pathologies or traumatic injuries, thereby preventing unnecessary invasive procedures, especially in clinically stable patients [3].

Regarding clinical and histopathological characteristics, HHs are the most prevalent benign hepatic neoplasms, often detected incidentally during imaging studies performed for unrelated medical issues [4]. Histologically, they consist of blood-filled vascular channels lined by endothelial cells [5]. Clinically, they are usually asymptomatic and require no therapeutic intervention except in cases of giant size or when symptomatic [6]. In contrast, HAPs are rare, acquired vascular lesions resulting from a breach in the arterial wall, leading to a contained blood-filled cavity. They frequently arise following trauma, surgical interventions, or inflammatory processes. Clinically, HAPs may present with nonspecific symptoms such as abdominal pain or jaundice, especially if compressing adjacent structures or rupturing, and they most often mandate invasive management [7].

Concerning imaging features, typical HHs demonstrate peripheral nodular enhancement with progressive centripetal fill-in on delayed phases. A subset known as “flash-filling hemangiomas” shows rapid, homogeneous enhancement during the arterial phase and retains contrast without washout in later phases [6]. In contrast, HAPs appear as well-defined, intensely enhancing lesions that mirror arterial blood pool densities across all phases, reflecting their vascular nature [7].

B-mode ultrasound (US) is typically employed as the initial imaging modality for HHs, where it often presents as well-defined, hyperechoic lesions with posterior acoustic enhancement. The diagnostic performance of US, however, is strongly influenced by lesion size: sensitivity is reported at only 17–20% for lesions smaller than 1 cm, improves to 65–80% for those measuring 1–2 cm, and approaches nearly 100% for larger lesions. In cases with atypical features, contrast-enhanced ultrasound (CEUS) provides significant added value, being rapid, non-invasive, and cost-effective. CEUS features follow the MRI and CT with contrast features. It enables correct diagnosis in approximately 85% of cases within 30 min, with fewer than 15% requiring further evaluation with CT or MRI. Its reported diagnostic accuracy is excellent, with sensitivity of 90–95% and specificity of 99–100%, particularly for lesions greater than 2 cm [8]. In contrast, the diagnostic yield of US and Doppler US for HAP is more limited: while specificity is high, sensitivity is modest at around 37.5%, with small or deeply located lesions frequently missed. CEUS markedly improves detection in this setting, increasing sensitivity to 75%, maintaining 100% specificity, and achieving nearly 99.9% diagnostic accuracy, comparable to CT angiography in post-transplant patients. Furthermore, in traumatic abdominal settings, CEUS has shown excellent performance in the evaluation of vascular injuries, with sensitivity of ~ 97% and specificity of ~ 88%, underlining its critical role in detecting even small or otherwise inconspicuous HAPs [9].

In our case, the patient underwent abdominal dynamic CT with contrast, which revealed a small (7 mm) cystic, blood-filled cavity showing arterial phase enhancement. This raised concern for a possible vascular injury (HAP), prompting the decision to proceed with Transarterial embolization (TAE). However, during the procedure, the lesion demonstrated the characteristic “flush” appearance of HH, leading to the abortion of embolization. Subsequent MRI confirmed the diagnosis, with features consistent with HH. In this context, US or CEUS were of limited utility given the diminutive lesion size and the acute trauma setting.

The first diagnostic dilemma arises with flash-filling HHs, since their arterial phase enhancement closely mimics HAPs. Both lesions may appear as hypervascular masses with intense arterial uptake, making early-phase imaging alone unreliable [10]. The distinction rests on clinical context, lesion morphology, and delayed-phase enhancement: HAPs typically retain arterial density throughout all phases, whereas HHs, despite rapid arterial enhancement, usually retain contrast without washout [11].

A further challenge was the small size of the lesion, which complicated differentiation. Lesions < 2 cm frequently show atypical imaging features that overlap between HH and HAP [12]. Flash-filling HHs, often < 2 cm, demonstrate rapid, homogeneous arterial enhancement, mimicking HAPs [13]. This similarity, combined with small lesion size, creates significant diagnostic uncertainty.

Regarding management, TAE is the standard of care for HAPs, particularly in polytrauma cases such as our patient. However, once HH was suspected intra-procedurally, the intervention was rightly aborted, and the pathology reassessed with further imaging. Although percutaneous or endovascular procedures such as ablation or embolization can be used for HH, these are rarely applied and generally reserved for giant or symptomatic cases [14, 15].

Finally, other differential diagnoses of hypervascular hepatic lesions encompass a range of benign and malignant entities which are crucial to be considered. For example, focal nodular hyperplasia (FNH) is typically seen in young women, often incidental, and is characterized by a central scar with homogeneous arterial enhancement and isoattenuation on delayed phases. Furthermore, hepatocellular adenoma (HCA), usually linked to oral contraceptive or anabolic steroid use, shows arterial phase hyperenhancement but often demonstrates fat, hemorrhage, or heterogeneous signal intensity, lacking the central scar of FNH. Moreover, hepatocellular carcinoma (HCC) arises most frequently in cirrhotic livers, with hallmark arterial phase hyperenhancement followed by washout in portal or delayed phases. In addition, hypervascular metastases (e.g., from neuroendocrine tumors, renal cell carcinoma, or melanoma) present as multiple lesions with early intense enhancement and variable washout, usually in patients with a known primary malignancy. Clinical context, risk factors, and multiphasic imaging features are therefore critical for accurate distinction among these entities [16]. Although, they were excluded in our case according to our clinical and imaging scenario.

## Conclusion

In emergency setting, this case emphasizes the importance of being aware of the specific characteristics which differentiates the hepatic haemangiomas from other vascular lesions or traumatic injuries as pseudoaneurysms through additional accurate non-invasive such as CT or MRI which can delineate the lesion's features and distinguish it from other hepatic pathologies or traumatic injuries to prevent unnecessary invasive procedures especially in clinically stable patient.

## Data Availability

The data that support the findings of this study are available upon request from the authors at (Department of Vascular and Interventional Radiology, Image-Guided Therapy Center, François-Mitterrand University Hospital, Dijon, France).

## Abbreviations

HAP: Hepatic artery pseudoaneurysm
HH: Hepatic haemangioma
CT: Computed tomography
TAE: Trans-arterial embolization
MRI: Magnetic resonance imaging
AST: Aspartate aminotransferase
ALT: Alanine aminotransferase
CA: Celiac artery
RHA: Right hepatic artery
MIP: Maximum intensity projection
